# Impact of a decision aid about stratified ovarian cancer risk-management on women’s knowledge and intentions: a randomised online experimental survey study

**DOI:** 10.1186/s12889-017-4889-0

**Published:** 2017-11-16

**Authors:** Susanne F. Meisel, Maddie Freeman, Jo Waller, Lindsay Fraser, Sue Gessler, Ian Jacobs, Jatinderpal Kalsi, Ranjit Manchanda, Belinda Rahman, Lucy Side, Jane Wardle, Anne Lanceley, Saskia C. Sanderson, on behalf of the PROMISE team

**Affiliations:** 1 Department of Behavioural Science and Health, University College London, London, UK; 2 Institute of Psychiatry, Psychology and Neuroscience, King’s College London, London, UK; 3 Department of Women’s Cancer, Institute for Women’s Health, University College London, London, UK; 4 University of New South Wales, Sydney, NSW Australia; 5 Great Ormond Street Hospital, London, UK; 6 University College Southampton, Southampton, UK

## Abstract

**Background:**

Risk stratification using genetic and other types of personal information could improve current best available approaches to ovarian cancer risk reduction, improving identification of women at increased risk of ovarian cancer and reducing unnecessary interventions for women at lower risk. Amounts of information given to women may influence key informed decision-related outcomes, e.g. knowledge. The primary aim of this study was to compare informed decision-related outcomes between women given one of two versions (gist vs. extended) of a decision aid about stratified ovarian cancer risk-management.

**Methods:**

This was an experimental survey study comparing the effects of brief (gist) information with lengthier, more detailed (extended) information on cognitions relevant to informed decision-making about participating in risk-stratified ovarian cancer screening. Women with no personal history of ovarian cancer were recruited through an online survey company and randomised to view the gist (*n* = 512) or extended (*n* = 519) version of a website-based decision aid and completed an online survey. Primary outcomes were knowledge and intentions. Secondary outcomes included attitudes (values) and decisional conflict.

**Results:**

There were no significant differences between the gist and extended conditions in knowledge about ovarian cancer (time*group interaction: F = 0.20, *p* = 0.66) or intention to participate in ovarian cancer screening based on genetic risk assessment (t(1029) = 0.43, *p* = 0.67). There were also no between-groups differences in secondary outcomes. In the sample overall (*n* = 1031), knowledge about ovarian cancer increased from before to after exposure to the decision aid (from 5.71 to 6.77 out of a possible 10: *t* = 19.04, *p* < 0.001), and 74% of participants said that they would participate in ovarian cancer screening based on genetic risk assessment.

**Conclusions:**

No differences in knowledge or intentions were found between women who viewed the gist version and women who viewed the extended version of a decision aid about risk-stratified ovarian cancer screening. Knowledge increased for women in both decision aid groups. Further research is needed to determine the ideal volume and type of content for decision aids about stratified ovarian cancer risk-management.

**Trial registrations:**

This study was registered with the ISRCTN registry; registration number: ISRCTN48627877.

**Electronic supplementary material:**

The online version of this article (10.1186/s12889-017-4889-0) contains supplementary material, which is available to authorized users.

## Background

Ovarian cancer remains the leading cause of gynaecological cancer death in the UK, with approximately 11 women dying from the disease each day [1]. A lack of disease-specific symptoms during early disease makes diagnosis challenging and most women are diagnosed at an advanced stage when the cancer has already spread beyond the ovaries. Diagnosis at an earlier stage (e.g. Stage 1) carries a significantly better prognosis with more than 90% of patients alive at 5 years post-diagnosis.

This observation has prompted a significant global effort to develop strategies that can lead to early detection of ovarian cancer. There is some evidence suggesting that a screening strategy involving serial sampling (annual or 4-monthly) of serum CA125 levels interpreted by the Risk of Ovarian Cancer Algorithm (ROCA), and incorporating selective transvaginal ultrasound scanning (TVS) may improve risk management of women in both the general population and those at increased risk [2–4]; however, the potential impact on disease-specific mortality is not yet known. The largest of the randomised controlled ovarian cancer screening trials, United Kingdom Collaborative Trial of Ovarian Cancer Screening (UKCTOCS), is continuing to follow its cohort to determine the long term impact of annual screening on ovarian cancer death rates [5]. At present screening for ovarian cancer using existing methodologies is not advocated [6].

The recent advances in genomic and other -omic technologies present an opportunity to develop more personalised approaches to cancer screening where genetic predisposition, epidemiological and lifestyle factors may be combined to assess individual cancer risk. Screening efficiency may be improved by focussing on high-risk individuals and tailoring screening and other preventative and early detection activities to their needs while minimising harms to individuals at lower disease risk. General population surveys and surveys of specific patient subgroups such as women at high risk of cancer suggest that public interest in stratified approaches to cancer risk-management is high [7–10].

As in almost all areas of medical care, patients must be helped to make decisions that reflect their autonomy and that are adequately informed [11, 12]. In addition to the usual anticipated harms of screening (including false-positive results, surgical complications, short-term anxiety, and overdiagnosis), identification of increased genetic risk may also prompt life-changing decisions to be made (e.g. prophylactic surgery) and may necessitate testing of family members with far reaching consequences. There is also the added complication of how to manage genetic variants of uncertain clinical significance. Patients will need to be helped to make informed decisions that take these complex potential benefits and risks into account and that are *‘based on relevant knowledge, consistent with the decision-maker’s values and behaviourally implemented’* [13].

Decision aids are evidence-based tools that outline different healthcare options and consequences in an organised fashion with the purpose of helping patients make informed decisions [14]. As well as conveying important information, decision aids also include questions aimed at clarifying personal values and preferences to remind the decision-maker to take these into account [15].

We are engaged in PROMISE (‘Predicting Risk of Ovarian Malignancies, Improved Screening and Early Detection’), a large collaborative international research programme that aims to develop and validate models for ovarian cancer risk stratification, early detection and diagnosis, incorporating clinical, epidemiological, proteomic and genetic data (https://www.eveappeal.org.uk/about/research/promise, accessed 28 March 2017). In PROMISE, we are also conducting a pilot study to investigate the feasibility of applying a stratified approach to managing ovarian cancer risk. To aid potential recruits to make a decision about participation in the pilot study applying the risk stratification approach developed in PROMISE, we have been working to develop a decision aid. As a first step in this process, we examined the impact of two beta versions of a decision aid that focused on the use of genetic testing in ovarian cancer risk stratification. We measured outcomes related to informed decision-making among people in the general population, and compared two levels of detail of the information presented.

We are not aware of any previous research addressing decisions about stratified cancer risk-management in a general population sample. Nor are we aware of any studies that have systematically evaluated how the volume of information contained in a decision aid affects cognitions relevant to informed decision-making for ovarian cancer risk-management. Therefore, we tested the impact of providing two levels of information (gist vs. extended) on cognitions relevant to informed decision-making about personalized (genetic) stratified ovarian cancer risk-management in a web-based experimental study.

### Study objectives and hypotheses

The primary research objective was to describe and compare key informed decision-related outcomes between two versions of a decision aid about personalized risk-stratified ovarian cancer screening (gist vs. extended). The primary outcomes were (1) **knowledge** about ovarian cancer and the PROMISE study and (2) **intention** to participate in personalized risk-stratified ovarian cancer screening in PROMISE; secondary outcomes included (3) **attitudes** (values) towards taking part in PROMISE. Based on cognitive load theory [16, 17], we hypothesised that participants receiving the shorter ‘gist’ information would have higher knowledge about key components of risk-stratified ovarian cancer screening in the PROMISE study than those receiving the longer ‘extended’ information. This is because fewer demands would be placed on working memory in the ‘gist’ condition and so content-related information would be processed more effectively. The secondary research objectives were to describe and compare attitudes (values) and other decision-related outcomes (**decisional conflict** and **decision satisfaction**) between the gist and extended versions of the information.

## Methods

### Study design

This was a single-blind, two-arm, individually-randomised, controlled experimental survey study comparing the effects of brief (gist-based) information with lengthier, more detailed (extended) information on cognitions relevant to informed choice about participation in personalized risk-stratified ovarian cancer screening (i.e. intentions, knowledge, attitudes). Given that an increasing amount of health information is accessed online, the study was web-based.

#### Decision aid

We created two versions of an online decision aid for the purpose of this study. The decision aid content was informed by: interviews about knowledge and attitudes towards the proposed PROMISE programme among members of the public; interviews about knowledge and attitudes towards the proposed PROMISE programme among women at high risk of ovarian cancer [10]; a systematic review of the literature; and existing clinical information designed for women at high risk of ovarian cancer. The decision aids outlined information about ovarian cancer risk, genetic testing, and the risk-stratification programme (PROMISE), and contained a values clarification exercise which aimed to help participants make a hypothetical decision about PROMISE that was in line with their personal values. The International Patient Decision Aid Standards Collaboration (IPDAS) criteria were followed and all content was written for grade 8 reading level or below, as recommended by IPDAS. One version of the decision aid contained gist information and the other contained extended information (see below). With the exception of amount of content detail (gist vs extended), all other components of the online decision aid (layout, overarching themes, and values clarification exercise) were standardised and the same in both versions. The content of both decision aids can be found in the Additional file 1.

#### Gist information

The ‘gist’ version of the decision aid was 5 pages long. Participants randomized to the gist version of the decision aid were invited to browse it at their leisure. They could access each component of the website in the order they preferred. At any point they could choose to access the values clarification exercise (by clicking on a ‘Ready to decide?’ button).

#### Extended information

The ‘extended’ version of the decision aid was 8 pages long, and was based on information given to patients attending clinical genetics services (designated as the ‘usual care’ arm of the trial). Participants randomized to the ‘extended’ version of the decision aid were also invited to browse it at their leisure. Identical to the intervention group, they could also access each component of the website in the order they preferred, and they could choose to access the values clarification exercise at any point.

### Recruitment

#### Participants

We included women aged 18–74 years. Quotas were set for education to broadly reflect those of the UK general population. Participants were recruited through an online survey company (Survey Sampling International; SSI) who were blind to the study hypothesis. SSI curates a panel of members of the UK general population who are willing to participate in online surveys in exchange for minor rewards (e.g. air miles), with the aim of reducing the possibility of drop-out and subsequent selection bias. Panel members invited to participate were asked whether they had ever been diagnosed with ovarian cancer in order to assess eligibility to complete the full survey. We excluded women with a *personal* history of ovarian cancer (but not breast cancer) because they were already able to access specific risk management options according to NICE (National Institute for Health and Care Excellence) guidelines [18]. After discussion with the wider research team, we decided not to exclude participants with a *family* history of ovarian cancer because it would be unlikely that they would be excluded if a stratified approach to ovarian cancer risk-management was introduced as a novel screening programme. Interested and eligible participants indicated their consent to take part in the study by entering and completing the baseline survey. It was made clear that data would be stored according to data protection regulations and that participants could withdraw from the study at any time without any personal consequences. It was made clear that the decision to participate in PROMISE was hypothetical only (i.e. they would not actually be able to receive information about their personal risk of developing ovarian cancer).

#### Study duration

Participants received the follow-up questionnaire via an email link 1–2 h after they accessed the website. Recruitment into the study stopped once target numbers were achieved. Data were collected in August 2015.

#### Randomisation

Consenting participants were randomised to the gist information group or the extended information group using an in-built randomisation algorithm used by SSI (group allocation ratio 1:1). Participants were blinded to their group allocation and were simply told they would be viewing one version of a website about an ovarian cancer screening study. All data, including randomisation, were recorded, minimising the risk of experimenter bias. Recording all data online also minimised the risk of missing data or inadvertent manipulation.

#### Sample size and assumptions

A power calculation using GPower (version 3.1) showed that a sample of 990 women would give 80% power to detect a between-group difference in informed decision-making outcomes (medium effect size) at alpha = 0.05.

#### Ethics

This study received ethical approval from UCL Research Ethics Committee.

### Measures

#### Demographic and personal information

*Demographic information* collected included age, ethnicity and education. Personal information included blood relatives diagnosed with ovarian cancer or breast cancer, past participation in any of the NHS cancer screening programmes (cervical, breast, colorectal), and perceived risk of ovarian [breast] cancer in comparison with other women of the same age (‘compared with other women your age would you say your risk of ovarian [breast] cancer is’…‘*much lower than others’, ‘lower than others’, ‘same as others’, ‘higher than others’, ‘much higher than others’*). This was based on a measure of perceived health risks [19]. Worry about ovarian cancer and worry about breast cancer were assessed with items used in previous studies: ‘I worry about getting ovarian [breast] cancer’ (‘*never’, ‘rarely’, ‘sometimes’, ‘often’, ‘most of the time’*). This measure was adapted from the Health Information National Trends Survey [20].

*Genetic literacy* was assessed with six statements used in previous research [21–24]: 1) Genes are inside cells (**true**); 2) Genes are made up of DNA (**true**); 3) Most genetic disorders are caused by a single gene (**not true)**; 4) If a person has a faulty gene that has been linked with a disorder, they will always get the disorder (**not true**); 5) On average, a person has half their genes in common with their biological siblings (**true**); 6) A mother and daughter who look alike have more genes in common than a mother and a daughter who do not look alike (**not true**). Response options for each question were *‘true’, ‘not true’, and ‘not sure’*.

#### Knowledge

*Knowledge about ovarian cancer* was assessed using four statements specifically developed for this study (bold font indicates response options coded as ‘correct’ and consistent with the information provided in the decision aid): 1) The average risk of a woman developing ovarian cancer in her lifetime is… (*1 in 8;* ***1 in 50*** *; 1 in 1000*); 2) Ovarian cancer mainly affects older women (***‘true’***; *‘not true’*; *‘not sure’)*; 3) Ovarian cancer can easily be found early with an ultrasound scan (*‘true’*; ***‘not true’***; *‘not sure’)*; 4) There are some factors that increase the risk of developing ovarian cancer: **being overweight; being over 50 years old; smoking; family history;** taking the pill; having children; breastfeeding; being inactive**;** using talcum powder; having IVF treatment (*Please tick all that you think apply)*. Participants also scored up to three points if they correctly identified that taking the pill, having children and breastfeeding were not risk factors for ovarian cancer, as these have clearly been shown to be protective factors. This gave a total possible score of 10. The questions were adapted from the ovarian cancer awareness measure toolkit [25].

*Knowledge about PROMISE* was assessed using six statements: 1) Genetic testing for ovarian cancer involves giving a blood sample (**true**); 2) PROMISE uses genetic information to estimate the risk of developing ovarian cancer (**true**); 3) Learning about personal risk of ovarian cancer may affect other family members (**true**); 4) Most women who take part in PROMISE will learn that they are at high risk of ovarian cancer (**not true**); 5) Women at high risk can’t do anything to reduce their chance of getting ovarian cancer (**not true**); 6) Women who take part in PROMISE will get some information about their risk of breast cancer (**true**). Response options for all statements were *‘true’; ‘not true’; ‘not sure’*.

#### Attitudes (values) towards taking part in PROMISE

Attitudes towards taking part in PROMISE were measured using a validated scale by Hersch and colleagues [26]. The scale consists of six items, each on a five-point response scale: Each item was preceded by the statement: ‘For me, taking part in ovarian cancer screening based on genetic risk assessment would be…’: A bad thing – Not a bad thing; Beneficial – Not beneficial; Harmful – Not harmful; A good thing – Not a good thing; Worthwhile – Not worthwhile; Important – Unimportant. Responses were summed and a mean attitude score was created ranging from 1 to 5.

#### Intention to participate in the PROMISE study

Intention to take part in risk-stratified ovarian cancer screening based on genetic risk was adapted from a question by Power and colleagues [27]: ‘If you were invited to take part in ovarian cancer screening based on genetic risk assessment would you take up the offer?*’*, with response options being *‘yes, definitely’; ‘yes, probably’; ‘probably not’; ‘definitely not’* (where 1 = definitely not, 4 = yes definitely)*.*

#### Decisional conflict and anticipated decisional regret

Decision-related outcomes were assessed using six items adapted from the decisional conflict scale [28]: 1) I would be satisfied with my decision of whether or not to take part in PROMISE, 2) I feel I would make an informed choice about taking part in PROMISE, 3) I would expect to stick with my decision of whether or not to take part in PROMISE, 4) I know the risks of taking part in PROMISE, 5) I feel unsure about what I would choose – taking part in PROMISE or not taking part, 6) The decision of whether or not to take part in PROMISE would be easy for me. Anticipated decisional regret was assessed using two items based on Abraham and Sheeran’s [29] measure of anticipated regret: 1) If I did not take part in PROMISE, I would feel regret; 2) If I did not take part in PROMISE, I would later wish I had. All responses were recorded on four-point scales ranging from strongly disagree to strongly agree. The decisional conflict items were reverse coded, so that a high score indicated high decisional conflict. A principal components analysis with varimax rotation was run and this confirmed the two factor structure (see Additional file 2: Table S1).

#### Website usage

Website usage was assessed at follow-up using three questions: ‘I read all the information on the website’ *(‘very thoroughly’; ‘quite thoroughly’; ‘briefly’; ‘just the parts I felt were relevant’*); ‘There was enough information on the website to make a decision about whether or not to take part in PROMISE (*‘strongly agree’; ‘agree’; ‘disagree’; ‘strongly disagree’*), and ‘I would have preferred to speak to a health professional before making a decision about whether or not to take part in PROMISE (*‘strongly agree’; ‘agree’; ‘disagree’; ‘strongly disagree’*).

Demographic and personal information, worry about breast and ovarian cancer, genetic literacy and health literacy were assessed before the decision aid intervention. Perceived risk of ovarian cancer and knowledge of ovarian cancer were measured pre and post exposure to the decision aid. Knowledge about PROMISE, attitudes, intentions, decisional conflict, anticipated decisional regret and website usage were assessed after the decision aid intervention only (see Table 1).

**Table 1:** Constructs measured and time of assessment

Construct	Time of assessment	
	Before exposure to decision aid	After exposure to decision aid
Demographic and personal information	✓	✗
Worry about breast and ovarian cancer	✓	✗
Genetic literacy	✓	✗
Health literacy	✓	✗
Perceived risk of ovarian cancer	✓	✓
Knowledge about ovarian cancer	✓	✓
Knowledge about PROMISE	✗	✓
Attitudes towards taking part in PROMISE	✗	✓
Intention to take part in PROMISE	✗	✓
Decisional conflict	✗	✓
Anticipated decisional regret	✗	✓
Website usage	✗	✓

### Statistical analyses

Socio-demographic and outcome variables were described using frequencies, means and standard deviations. Outcome variables were compared cross-sectionally between the ‘gist’ and ‘extended’ information decision aid conditions using a series of independent samples t-tests. Change over time in knowledge about ovarian cancer from before to after the decision aid was examined and compared between-groups using a repeated measures ANOVA with group (gist vs. extended) as an independent variable, and the time*group interaction reported. *P*-values of less than 0.05 were considered significant. All analyses were conducted with SPSS version 23.

## Results

### Sample characteristics

Overall, 1031 women participated in the study. There were 512 participants in the ‘gist’ decision aid group and 519 in the ‘extended’ group. Women were well distributed across the age groups, and ranged from 18 years upwards, with 7% aged 65 and over. The majority of participants were White (79.6%), spoke English as their first language (90.9%), and 26% were educated to Degree level. None had had a previous diagnosis of ovarian cancer. Twenty-five percent of participants had a family history of breast cancer, and 8.1% had a family history of ovarian cancer. Two thirds (68.8%) of participants perceived their risk of getting ovarian cancer to be the same as other women of their age. Forty-four percent of women reported worrying ‘sometimes’ about breast cancer, and 33.8% reported worrying ‘sometimes’ about ovarian cancer. The full socio-demographic characteristics of the sample and each group are illustrated in Table 2. In addition, (Additional file 2: Table S2) shows participants’ responses to the individual items for genetic literacy, and shows that participants’ mean genetic literacy score was 3.47 (where 0 = low and 6 = high).

**Table 2:** Socio-demographic characteristics of participants

	Total (n = 1031)	Gist group (*n* = 512)	Extended group (*n* = 519)
	*N* (%)	*N* (%)	*N* (%)
Age			
18–29	199 (19.3)	109 (21.3)	90 (17.3)
30–39	222 (21.5)	104 (20.3)	118 (22.7)
40–49	228 (22.1)	115 (22.5)	113 (21.8)
50–59	210 (20.4)	99 (19.3)	111 (21.4)
60+	172 (16.7)	85 (16.6)	87 (16.8)
Ethnicity			
White	821 (79.6)	402 (78.5)	419 (80.7)
Asian	95 (9.2)	52 (10.1)	43 (8.3)
Black	68 (6.6)	31 (6.0)	37 (7.1)
Mixed	34 (3.3)	20 (4.0)	14 (2.7)
Other	8 (0.8)	6 (1.2)	2 (0.4)
First language			
English	937 (90.9)	460 (89.8)	477 (91.9)
Other	93 (9.0)	52 (10.2)	41 (7.9)
Education			
Degree or equivalent	268 (26.0)	130 (25.4)	138 (26.6)
Higher education	112 (10.9)	55 (10.7)	57 (11.0)
A Level or equivalent	235 (22.8)	112 (21.9)	123 (23.7)
GCSEs grades A*-C or equivalent	256 (24.8)	140 (27.3)	116 (22.4)
Other qualifications	83 (8.1)	42 (8.2)	41 (7.9)
No formal qualifications	73 (7.1)	32 (6.3)	41 (7.9)
Family history of breast cancer (yes)	258 (25.0)	125 (24.4)	133 (25.6)
Family history of ovarian cancer (yes)	84 (8.1)	47 (9.2)	37 (7.1)
Comparative risk of ovarian cancer			
Much lower than others	79 (7.7)	37 (7.2)	42 (8.1)
Lower than others	147 (14.3)	81 (15.8)	66 (12.7)
Same as others	709 (68.8)	350 (68.4)	359 (69.2)
Higher than others	85 (8.2)	39 (7.6)	46 (8.9)
Much higher than others	11 (1.1)	5 (1.0)	6 (1.2)
Worry about breast cancer			
Never	102 (9.9)	43 (8.4)	59 (11.4)
Rarely	270 (26.2)	139 (27.1)	131 (25.2)
Sometimes	457 (44.3)	235 (45.9)	222 (42.8)
Often	152 (14.7)	70 (13.7)	82 (15.8)
Always	50 (4.8)	25 (4.9)	25 (4.8)
Worry about ovarian cancer			
Never	256 (24.8)	115 (22.5)	141 (27.2)
Rarely	354 (34.3)	184 (35.9)	170 (32.8)
Sometimes	348 (33.8)	177 (34.6)	171 (32.9)
Often	53 (5.1)	22 (4.3)	31 (6.0)
Always	20 (1.9)	14 (2.7)	6 (1.2)
Previous participation in cancer screening tests			
Cervical	774 (75.1)	377 (73.6)	397 (76.5)
Breast	400 (38.8)	193 (37.7)	207 (39.9)
Colorectal	162 (15.7)	79 (15.4)	83 (16.0)
None of above	214 (20.8)	114 (22.3)	100 (19.3)

*Note.* In cases where percentages do not add up to 100%, this represents missing data

### Knowledge

*Knowledge about ovarian cancer* increased from pre- to post-intervention in the sample overall (from 5.71 to 6.77, out of a possible 10: F = 362.25, *p* < 0.001). There was no significant difference between the gist and extended conditions (time*group interaction: F = 0.20, *p* = 0.66). The mean score for *knowledge about PROMISE* was 3.97 out of 6 after the decision aid; mean scores were not significantly different between the gist and extended conditions (4.02 vs. 3.92 respectively: t(1029) = 0.87, *p* = 0.39) (see Table 3). In addition, (Additional file 2: Table S3) shows participants’ knowledge in the sample overall.

**Table 3:** Knowledge about ovarian cancer and PROMISE, attitudes and intentions compared between the gist and extended decision aid groups

		Pre decision-aid		Post decision-aid		P-value for difference between groups
	Response options	Gist group (n = 512) N (%)	Extended group (n = 519) N (%)	Gist group (n = 512) N (%)	Extended group (n = 519) N (%)	
Knowledge						
Summary scores						
Knowledge about ovarian cancer (range = 0–10), mean (SD)		5.75 (1.63)	5.66 (1.48)	6.79 (1.84)	6.76 (1.81)	*t*(1029) = 0.30, *p* = 0.766
Knowledge about PROMISE (range = 0–6), mean (SD)		n/a	n/a	4.02 (1.73)	3.92 (1.70)	*t*(1029) = 0.87, *p* = 0.386
Individual item scores						
*Knowledge about ovarian cancer:*						
The average risk of a woman developing ovarian cancer in her lifetime is (1 in 50)	1) 1 in 8 **2) 1 in 50** 3) 1 in 1000	141 (27.5) **280 (54.7)** 91 (17.8)	144 (27.7) **256 (49.3)** 119 (22.9)	117 (22.9) **347 (67.8)** 48 (9.4)	105 (20.2) **346 (66.7)** 68 (13.1)	
Ovarian cancer mostly affects older women (True)	**True** Not true Not sure	**106 (20.7)** 320 (62.5) 86 (16.8)	**85 (16.4)** 330 (63.6) 104 (20.0)	**246 (48.0)** 203 (39.6) 63 (12.3)	**211 (40.7)** 238 (45.9) 70 (13.5)	
Ovarian cancer can easily be found early with an ultrasound scan (Not true)	True **Not true** Not sure	238 (46.5) **89 (17.4)** 185 (36.1)	233 (44.9) **85 (16.4)** 201 (38.7)	211 (41.2) **124 (24.2)** 177 (34.6)	210 (40.5) **147 (28.3)** 162 (31.2)	
There are some factors which increase the risk of ovarian cancer. Please tick all that you think apply:						
Being over 50 years old (Yes)	**Yes** No	**222 (43.4)** 290 (56.6)	**213 (41.0)** 306 (59.0)	**340 (66.4)** 172 (33.6)	**322 (62.0)** 197 (38.0)	
Being overweight (Yes)	**Yes** No	**270 (52.7)** 242 (47.3)	**285 (54.9)** 234 (45.1)	**342 (66.8)** 170 (33.2)	**356 (68.6)** 163 (31.4)	
Smoking (Yes)	**Yes** No	**293 (57.2)** 219 (42.8)	**294 (56.6)** 225 (43.4)	**344 (67.2)** 168 (32.8)	**351 (67.6)** 168 (32.4)	
Family history (Yes)	**Yes** No	**420 (82.0)** 92 (18.0)	**436 (84.0)** 83 (16.0)	**413 (80.7)** 99 (19.3)	**445 (85.7)** 74 (14.3)	
Taking the pill (No)	Yes **No**	197 (38.5) **315 (61.5)**	197 (38.0) **322 (62.0)**	150 (29.3) **362 (70.7)**	162 (31.2) **357 (68.8)**	
Having children (No)	Yes **No**	67 (13.1) **445 (86.9)**	58 (11.2) **461 (88.8)**	53 (10.4) **459 (89.6)**	50 (9.6) **469 (90.4)**	
Breastfeeding (No)	Yes **No**	9 (1.8) **503 (98.2)**	16 (3.1) **503 (96.9)**	12 (2.3) **500 (97.7)**	16 (3.1) **503 (96.9)**	
*Knowledge about PROMISE:*						
Genetic testing for ovarian cancer involves giving a blood sample (True)	**True** Not true Not sure	n/a	n/a	**279 (54.5)** 67 (13.1) 166 (32.4)	**282 (54.3)** 69 (13.3) 168 (32.4)	
PROMISE uses genetic information to estimate the risk of developing ovarian cancer (True)	**True** Not true Not sure	n/a	n/a	**360 (70.3)** 29 (5.7) 123 (24.0)	**357 (68.8)** 21 (4.0) 141 (27.2)	
Learning about personal risk of ovarian cancer may affect other family members (True)	**True** Not true Not sure	n/a	n/a	**383 (74.8)** 43 (8.4) 86 (16.8)	**392 (75.5)** 56 (10.8) 71 (13.7)	
Most women who take part in PROMISE will learn that they are at high risk of ovarian cancer (Not true)	True **Not true** Not sure	n/a	n/a	61 (11.9) **336 (65.6)** 115 (22.5)	83 (16.0) **320 (61.7)** 116 (22.4)	
There is nothing that women who are at high risk of ovarian cancer can do to reduce their risk of ovarian cancer (Not true)	True **Not true** Not sure	n/a	n/a	53 (10.4) **337 (65.8)** 122 (23.8)	56 (10.8) **313 (60.3)** 150 (28.9)	
Women who take part in PROMISE will get some information about their risk of breast cancer (True)	**True** Not true Not sure	n/a	n/a	**361 (70.5)** 15 (2.9) 136 (26.6)	**372 (71.7)** 27 (5.2) 120 (23.1)	
Attitudes towards taking part in PROMISE						
For me, taking part in PROMISE would be:						
A bad thing (1) not a bad thing (5)		n/a	n/a	3.93 (0.91)	3.89 (0.86)	
Not beneficial (1) beneficial (5)		n/a	n/a	3.89 (0.94)	3.84 (0.90)	
Not a good thing (1) a good thing (5)		n/a	n/a	3.93 (0.91)	3.89 (0.88)	
Not worthwhile (1) worthwhile (5)		n/a	n/a	3.91 (0.95)	3.88 (0.93)	
Not important (1) important (5)		n/a	n/a	3.82 (1.0)	3.82 (0.94)	
Harmful (1) not harmful (5)		n/a	n/a	4.04 (0.88)	4.03 (0.86)	
Total mean (range = 1–5) (SD)		n/a	n/a	3.92 (0.83)	3.89 (0.79)	t(1029) = 0.60, *p* = 0.550
Intention to participate in PROMISE						
Definitely not		n/a	n/a	18 (3.5%)	20 (3.9%)	
Probably not		n/a	n/a	114 (22.3%)	114 (22.0%)	
Yes probably		n/a	n/a	255 (49.8%)	267 (51.4%)	
Yes definitely		n/a	n/a	125 (24.4%)	118 (22.7%)	
Mean score (range = 1–4) (SD)		n/a	n/a	2.95 (0.78%)	2.93 (0.77%)	t(1029) = 0.43, *p* = 0.671

*Note.* Correct responses are shown in bold

### Attitudes (values) towards participating in PROMISE

The mean score for attitudes towards PROMISE was 3.91 (where 1 = negative attitude, 5 = positive attitude); mean scores were not significantly different between the gist and extended conditions (3.92 vs. 3.89 respectively, t(1029) = 0.60, *p* = 0.55) (see Table 3).

### Intention to participate in the PROMISE study

In the sample overall, 74% of participants said that they ‘probably’ or ‘definitely’ would participate in ovarian cancer screening based on genetic risk assessment (the PROMISE study); there was no difference between the gist and extended conditions (t(1029) = 0.43, *p* = 0.67) (see Table 3).

### Decisional conflict and anticipated decision regret

*Decisional conflict:* Overall, the majority (94%) of participants agreed or strongly agreed that they would feel that they would be making an informed choice about taking part in PROMISE, and three quarters (77%) reported that they knew the risks of taking part in PROMISE. However, over half (54%) also felt unsure about what they would choose, and only two thirds (67%) felt that the decision about whether or not to take part in PROMISE would be easy for them. The mean score for decisional conflict was 2.09 (SD = 0.32) (where 1 = low decisional conflict, and 4 = high decisional conflict). Mean scores did not differ between the gist and extended information decision aid groups (t(1029) = 0.20, *p* = 0.84).

*Anticipated decisional regret:* In the sample overall, 49% of participants agreed or strongly agreed that if they did not take part in PROMISE they would feel regret, and similarly 55% agreed or strongly agreed that if they did not take part, they would later wish they had. The mean anticipated decisional regret score was 2.56 (SD = 0.66), on a scale where 1 = low anticipated regret, and 4 = high anticipated regret. Mean scores did not differ between the gist and extended information groups (t(1029) = 0.02, *p* = 0.98) (see Table 4).

**Table 4:** Decisional conflict and anticipated decisional regret

Item (1 = strongly disagree, 4 = strongly agree)	Overall (n = 1031)	Gist group (n = 512)	Extended group (n = 519)
Decisional conflict	Mean (SD) % Agree/Strongly agree		
I would be satisfied with my decision of whether or not to take part in PROMISE	1.91 (.51) 92.6	1.91 (.52) 91.6	1.90 (.49) 93.6
I feel I would make an informed choice about taking part in PROMISE	1.84 (.52) 94.4	1.83 (.52) 94.5	1.85 (.51) 94.2
I know the risks of taking part in PROMISE	2.10 (.67) 76.9	2.09 (.67) 77.3	2.11 (.67) 76.5
I feel unsure of what I would choose - taking part in PROMISE or not taking part	2.53 (.76) 53.6	2.57 (.78) 52.0	2.48 (.74) 55.3
The decision about whether or not to take part in PROMISE would be easy for me	2.22 (.70) 67.4	2.18 (.71) 69.5	2.25 (.70) 65.3
I would expect to stick with my decision of whether or not to take part in PROMISE	1.93 (.54) 90.5	1.94 (.56) 89.5	1.91 (.52) 91.5
Mean subscale score (range = 1–4) (SD)	2.09 (0.32)	2.09 (0.32)	2.08 (0.32)^a^
Anticipated decisional regret			
If I did not take part in PROMISE, I would feel regret	2.52 (.72) 49.3	2.50 (.71) 48.6	2.54 (.72) 49.9
If I did not take part in PROMISE, I would later wish I had	2.60 (.72) 54.8	2.62 (.71) 56.7	2.57 (.73) 52.9
Mean subscale score (range = 1–4) (SD)	2.56 (0.66)	2.56 (0.66)	2.56 (0.67)^b^

^a^No significant difference between groups: *t*(1029) = 0.203, *p* = 0.839

^b^No significant difference between groups: *t*(1029) = 0.020, *p* = 0.984

### Website usage

There was no significant difference between the gist and extended groups in the amount of information on the website-based decision aid that participants reported reading (t(1029) = −0.15, *p* = 0.88). There was also no significant difference between the two groups in the proportions of participants who thought the website contained sufficient information to make a decision about whether or not to take part in PROMISE (t(1029) = −1.34, *p* = 0.18) (see Table 5).

**Table 5:** Website usage

Item	Response options	Total sample (n = 1031) *N* (%)	Gist group (n = 512) *N* (%)	Extended group (n = 519) *N* (%)
I read all the information on the website	Very thoroughly Quite thoroughly Briefly Just the relevant parts	216 (21.0) 446 (43.3) 268 (26.0) 101 (9.8)	100 (19.5) 233 (45.5) 134 (26.2) 45 (8.8)	116 (22.4) 213 (41.0) 134 (25.8) 56 (10.8)
Mean (range = 1–4) (SD)		2.25 (0.90)	2.24 (0.87)	2.25 (0.92)^a^
Imagine you were invited to take part in the study described on the website when you answer the following questions. There was enough information on the website to make a decision whether or not to take part in the study.	Strongly agree Agree Disagree Strongly disagree	281 (27.3) 679 (65.9) 64 (6.2) 7 (0.7)	141 (27.5) 345 (67.4) 24 (4.7) 2 (0.4)	140 (27.0) 334 (64.4) 40 (7.7) 5 (1.0)
Mean (range = 1–4) (SD)		1.80 (0.57)	1.78 (0.54)	1.83 (0.60)^b^

^a^No significant difference between groups: t(1029) = −0.149, *p* = 0.882

^b^No significant difference between groups: t(1029) = −1.336, *p* = 0.182

## Discussion

This study examined the effects of a web-based decision aid on key components of informed decision-making about risk-stratified ovarian cancer management, and compared the effects between a gist and an extended version of the decision aid. We found that knowledge about ovarian cancer improved significantly overall, indicating that the decision aid was successful in increasing knowledge; an essential component of informed choice. This finding is consistent with a review which found that decision aids increased patients’ knowledge about treatment or screening options [30]. Although scores on most of the ovarian cancer knowledge items were high after participants had viewed the decision aid, two of the items (‘Ovarian cancer mostly affects older women’ [True], and ‘Ovarian cancer can be found easily with an ultrasound scan’ [Not true]) were coded as being answered correctly by fewer than half the participants (44.3% and 26.3% respectively). This may reflect ambiguity in the question wording, and/or that the decision aids did not express this information sufficiently clearly. Future versions of the information presented to participants in the decision aid and the measures used to assess understanding of that information will need to make these facts and their measurement clearer.

To our knowledge, this is the first study to assess the impact of a gist versus extended version of a decision aid about risk-stratified ovarian cancer screening on aspects of informed decision-making. Different components of informed decision-making were measured, including knowledge, intentions and attitudes, in an attempt to capture the multidimensional nature of informed choice [13]. Moreover, individual item scores were examined, rather than just their composites, to obtain a detailed understanding of participants’ knowledge and beliefs. The study sample was large and diverse in terms of age and education. Despite the lack of difference between the two decision aids on informed choice outcomes, it was encouraging to observe that more participants intended to participate in PROMISE than not (74.2% vs. 25.8%). This suggests that a risk-stratified approach to screening for ovarian cancer and other management options might be considered acceptable to the majority of women within the general public, and if it were to be introduced, uptake could be high.

Our findings also suggest that providing participants in the planned PROMISE feasibility study with a decision aid may valuably help increase potential participants’ understanding of some key concepts, and that either a gist or extended version could achieve this desired outcome. There were no differences in knowledge about ovarian cancer, knowledge about the PROMISE study or intentions to participate in the PROMISE programme between those who viewed the gist and those who viewed the extended version of the decision aid. Neither were there differences in attitudes towards PROMISE, anticipated regret or decisional conflict between the two groups. The absence of differences in components of informed decision-making between the two groups suggests that the amount of information provided in the decision aid had little impact on people’s knowledge acquisition, attitudes or intention. Contrary to the study hypotheses, we found no evidence that participants in the gist group were able to retain more information, nor that those in the extended group experienced information overload. These findings support previous research which found no difference in colorectal cancer screening interest between participants who received a brief decision aid with two screening options and those who were given a more detailed decision aid containing five options [31].

An alternative explanation for the study’s results is that the two versions of the decision aid were not sufficiently different to elicit differences in informed decision-making outcomes. The gist version was five pages long, and the extended version was eight pages, so perhaps this was not enough of a difference to affect information-processing. In light of this it might be worthwhile for future research comparing different levels of information in decision aids to ensure that gist versions are limited to one or two pages, to establish a sufficient difference between experimental groups. Moreover, all participants were able to access as much or as little information as they desired, albeit within the confines of their version of the decision aid. Detail about which pages each participant accessed and for how long was not recorded, so it is not known how much information each participant actually read. It is therefore possible that the amount of information read by participants in the two groups did not differ.

It is also possible that the modality of the decision aid (i.e. website) was not particularly well-suited to the type of decision that was being made. Wilson and Wolf [17] claim that the type of medium chosen for a decision aid can affect cognitive load, which can impact upon informed decision making outcomes. Although static media, such as the decision aids used in the present study, are appropriate for certain medical contexts, e.g. when complex information must be repeatedly reviewed, video is potentially more effective for single-event decisions, e.g. deciding whether or not to take part in the PROMISE programme.

Decision aids are an effective tool to increase patients’ knowledge of their options when faced with a healthcare decision, so are likely to be valuable tools to help people make informed decisions. It is therefore potentially important that healthcare professionals consider providing patients with decision aids, and that such decision aids are available to them. This will be particularly important if stratified ovarian cancer risk-management becomes implemented in clinical practice, because it is a novel approach to screening and thus most patients’ knowledge of this concept will be low. Decision aids may be pivotal in improving knowledge levels and helping women to clarify their values in order to make informed decisions. Future research could explore the effect of providing women with decision aids about stratified ovarian cancer risk-management that are more different to each other in terms of the amount of information that is provided. The format of decision aid could also be manipulated, for example by using mobile apps and printed materials as well as websites, to ascertain whether this affects informed decision making outcomes.

## Conclusion

This study found that a web-based decision aid about (genetic) risk-stratified ovarian cancer screening significantly increased women’s knowledge about ovarian cancer. No differences in knowledge or other components of informed decision-making between participants who viewed a gist version vs. an extended version of the decision aid were observed. Further research is needed to determine the ideal volume and type of content for decision aids about stratified ovarian cancer risk-management.

## Additional files

Additional file 1: Decision aid text. The text that was included in the two versions of the Decision Aid used in the online experiment: 1. Gist version (pages 1 to 4); 2. Extended version (pages 5 to 10). (PDF 143 kb)

Additional file 2: Table S1. Factor loadings for decisional conflict and anticipated decisional regret items; **Table S2.** Participants’ knowledge about genetics (‘genetic literacy’); **Table S3.** Participants’ knowledge about ovarian cancer and the PROMISE study in the sample overall. (DOCX 16 kb)
