# Supplementary material for: Impact of a decision aid about stratified ovarian cancer risk-management on women’s knowledge and intentions: a randomised online experimental survey study
Source: BMC Public Health. 2017 Nov 16;17:882. doi: 10.1186/s12889-017-4889-0 (PMC5689140; doi:10.1186/s12889-017-4889-0)
Supplement: Supplementary file 1 — Decision aid text. The text that was included in the two versions of the Decision Aid used in the online experiment: 1. Gist version (pages 1 to 4); 2. Extended version (pages 5 to 10). (PDF 143 kb) [file 12889_2017_4889_MOESM1_ESM.pdf]

Additional File 1

Decision Aid: Gist version

Welcome

PROMISE is a new research study, jointly funded by Cancer Research UK and The Eve Appeal. All important information about the study can be found on this website. We invite you to browse the website before making a decision about whether or not to take part. You may read the information in any order, and you may read as much or as little as you like. Once you are ready to decide whether or not you would like to take part in PROMISE, please click on the red 'ready to decide' button featured on each page.

About Ovarian Cancer

- 1 in 50 women in the UK will develop ovarian cancer in their lifetime.
- Ovarian cancer mostly affects women over 50, but younger women can get it, too.
- It is hard to detect ovarian cancer early, which makes it more difficult to treat successfully.

Symptoms

Experts agree that the following symptoms are common in women with ovarian cancer:

- persistent pain in the tummy or lower part of the tummy
- increased tummy size or bloating
- feeling full quickly and/or loss of appetite
- needing to pass urine urgently and/or more often than usual.

Lots of women experience symptoms similar to these and usually they are nothing serious. But you should see your doctor if you regularly have any of these symptoms, which are not normal for you, and they happen on most days for three weeks or more. Other symptoms to look out for include:

- changes in bowel habit
- irregular bleeding or bleeding after the menopause
- extreme tiredness or back pain.

If you have any of the symptoms mentioned above, and they happen again and again, or they are new for you, it is important to see your doctor. If you have already been to your doctor and the symptoms have got worse or have not gone away, it is best to go back again and get checked out.

Risk Factors

Factors increasing the risk of ovarian cancer

- Being overweight
- Smoking
- Age
- Hormone replacement therapy (HRT)
- Family history

Factors decreasing the risk of ovarian cancer

- Having children
- Taking the Pill
- Breastfeeding

About Genetic Testing

Genes are the basic unit of heredity. They carry the information that determine the characteristics of a person. Genes act like a set of instructions, controlling our growth and how our bodies work.

Sometimes changes in genes can cause them not to work properly. In some cases this may increase a woman's risk of getting ovarian cancer at some point in her life.

Genetic testing involves giving a small sample of your blood to look for genetic changes which increase the risk of ovarian cancer.

About PROMISE

- Currently, it is difficult to detect ovarian cancer early.
- PROMISE is a research study. It will look at a woman's genes to estimate her personal lifetime risk of ovarian cancer.
- The results of genetic testing will be combined with other health information such as age and family history of breast and ovarian cancer to give a personal risk of ovarian cancer. This will allow health professionals to offer prevention and risk management options that are best suited to each individual woman.

What is the PROMISE study?

Currently, there are two options to manage a high risk of ovarian cancer: Screening, and surgery to remove the ovaries.

- Screening aims at detecting cancer early by looking at certain blood markers.
- Surgery will prevent the cancer from ever developing. However, removal of the ovaries means that the woman will go into menopause.

What happens next?

There are three possible outcomes of participating in this programme.

You may learn that you are at LOW, INTERMEDIATE or HIGH risk of ovarian cancer.

- Women in the LOW risk category will not need regular screening. Most women will be in this category. However, it is important to be aware of the symptoms of ovarian cancer.
- Women in the INTERMEDIATE and HIGH risk categories will be invited back to their GP to discuss options to reduce their risk. This may include screening or the offer of surgery to have their ovaries removed.

How is ovarian cancer risk assessed

It may be helpful to weigh up the potential benefits and risks before taking part in genetic testing for risk of ovarian cancer. It is your decision whether or not to have a genetic test to learn about your lifetime risk of ovarian cancer.

Benefits

- Genetic testing will help to estimate personal risk of ovarian cancer.
- The test result will give some information about breast cancer risk.

Potential Harms

- Receiving a high risk result may be upsetting.
- The genetic test will give some information about the risk of ovarian cancer for other women in your family.
- The test can only tell about a woman's lifetime risk, not whether she has cancer right now.
- Risk estimates aren't perfect. Genes are only one factor that influences a woman's risk of ovarian cancer.

Further information

- **Who is funding this study?** This study is currently jointly funded by Cancer Research UK and The Eve Appeal.
- **Will taking part in the study affect my life insurance?** Sometimes people worry that if they have a genetic test they will have problems taking out life insurance. There is an agreed code of practice among insurance companies that results of genetic testing will not be used by the Association of British Insurers (ABI) to determine premiums or eligibility for life insurance. Any genetic tests taken after the insurance policy has started do not have to be disclosed. This agreement was recently extended until 2017. Further information can be found on the website: www.abi.org.uk
- **I don't want to get a genetic test for risk of ovarian cancer. Can I still have ovarian cancer screening?** No, unfortunately not. Without the genetic test, health professionals cannot tell whether ovarian cancer screening would carry more risks than benefits for you. Therefore, you will have to have a genetic test first.
- **Why do none of the other screening programmes use genetic testing?** Although this is the first ovarian cancer screening study that includes genetic testing, it is likely that other cancer screening programmes will eventually also include genetic testing. For example, another research study is currently looking at genetic testing for breast cancer risk within the existing NHS Breast Screening Programme.
- **What can I do to reduce my risk of ovarian cancer?** You may be able to reduce your risk of developing ovarian cancer by making changes to your lifestyle.
- **Lifestyle and prevention advice: Weight and smoking** Maintaining a healthy weight may help to reduce risk of ovarian cancer. Smoking may also increase the risk of ovarian cancer.
- **Using Oral Contraceptives ('The Pill')** Using the oral contraceptive pill for at least five years may reduce risk of ovarian cancer by up to 50%. We would not recommend you use the pill for this specific reason but it is a beneficial side effect.
- **Confidentiality** Your participation in this study, and your results, will be completely confidential. Any information about yourself that you provide while taking part will be stored on a secure password protected database. This information will be treated as

confidential and not released to anyone outside the research team working on this study.

- **Who are the doctors and researchers involved in this study?** This study is carried out by health professionals under the leadership of Professor Ian Jacobs and coordinated by University College London. We are also working with researchers from the University of Cambridge, University of Manchester, University of Southern California, Massachusetts General Hospital and Harvard University.

Glossary

- **BRCA1 and BRCA2** are ‘cancer protection’ genes. Inherited mutations in these genes increase the risk of breast and ovarian cancer because the gene is not working properly.
- **CA125** is a protein found in the blood which may indicate the presence of ovarian cancer
- **Chromosomes** is DNA which is packaged into compact units
- **DNA** is the material that makes up genes and chromosomes
- **Genes** are made up of DNA and carry the information that determines a person’s features or characteristics
- **Genetic testing** is used to look for mutations (changes) in genes from a blood sample
- **Mutation** a permanent change to a gene that stops it from working properly
- **Menopause** is when a woman’s periods stop either naturally or through surgery or other treatments
- **Ovarian cancer** is cancer of the ovary
- **Ovary** is the part of the female reproductive system which contain the eggs
- **PROMISE** Predicting Risk of Ovarian Malignancies, Improved Screening and Early detection
- **Risk-reducing salpingo oophorectomy** is surgery to remove the fallopian tubes and ovaries
- **Screening** is testing individuals who have no symptoms to see if a disease is present or not

Decision Aid: Extended version

WELCOME

PROMISE is a new research study, jointly funded by Cancer Research UK and The Eve Appeal. All important information about the study can be found on this website. We invite you to browse the website before making a decision about whether or not to take part. You may read the information in any order, and you may read as much or as little as you like. Once you are ready to decide whether or not you would like to take part in PROMISE, please click on the red 'ready to decide' button featured on each page.

About Ovarian Cancer

Ovaries The ovaries are part of the female reproductive system. There is one on each side of a woman's lower tummy. Each is about the size and shape of an almond. Thousands of eggs are stored inside the ovaries. During childbearing years, an egg is released each month for a possible pregnancy. The ovaries also produce hormones which control monthly menstrual cycles.

Ovarian cancer The ovaries are made up of millions of cells. Some of these cells make hormones *(a hormone is a signalling molecule)*, some surround the eggs as they develop, and others make up the outside layer of the ovary. Ovarian cancer develops when cells of the ovaries start dividing out of control.

How often ovarian cancer occurs in the population Ovarian cancer is not very common. The lifetime risk of developing ovarian cancer is approximately 1 in 50 for UK women. Ovarian cancer mostly affects women over 50, but younger women can get it too.

Screening for ovarian cancer Ovarian cancer is very difficult to detect early. At the moment, there is no reliable screening test for ovarian cancer. Measuring a blood marker, called CA125 *(CA125 is a protein found in the blood which may show that a woman has ovarian cancer)*, and transvaginal ultrasound scans *(A transvaginal ultrasound scan is an ultrasound scan of the ovaries)* have been looked at more closely. However, these tests miss a lot of cases, and are therefore not very reliable for women who have not got a family history of ovarian cancer.

Factors increasing your risk for ovarian cancer

- **Weight** – Being overweight or obese increases the risk of ovarian cancer. Keeping a healthy weight may help to lower the chances of developing ovarian cancer.
- **Smoking** – Women who smoke are at higher risk of developing some types of ovarian cancer. Quitting smoking can help to cut the risk of ovarian cancer and other conditions.
- **Age** – As a woman gets older, her risk of developing ovarian cancer increases. More than eight in 10 cases are in women over 50.
- **HRT** – Using hormone replacement therapy (HRT), particularly oestrogen-only HRT, can increase a woman's risk of ovarian cancer. The longer women take HRT, the more the risk may increase. Once HRT is stopped this risk goes down over a few years to the same level as that of women who have never taken HRT.
- **Family history** – Women with a family history of ovarian or breast cancer may have an increased risk of ovarian cancer. About one woman in 10 who develops ovarian cancer is born with a faulty gene that runs in her family. But having a relative with ovarian cancer does not always mean you have a faulty gene. If you think you have a family history of ovarian cancer, ask your doctor for more information and advice.

Factors decreasing your risk for ovarian cancer

- **Having children** – The more children a woman has, the lower her risk of ovarian cancer.
- **Taking the Pill** – Oral contraceptives, also known as the Pill, significantly reduce the chance of developing ovarian cancer, both while they are taken and long after a woman stops. Taking the Pill slightly increases the risk of breast cancer, but only while it is taken.
- **Breastfeeding** – Women who breastfeed their children may have a lower risk of ovarian cancer.

Symptoms

Experts agree that the following symptoms are common in women with ovarian cancer:

- persistent pain in the tummy or lower part of the tummy
- increased tummy size or bloating
- feeling full quickly and/or loss of appetite
- needing to pass urine urgently and/or more often than usual.

Lots of women experience symptoms similar to these and usually they are nothing serious. But you should see your doctor if you regularly have any of these symptoms, which are not normal for you, and they happen on most days for three weeks or more. Other symptoms to look out for include:

- changes in bowel habit
- irregular bleeding or bleeding after the menopause
- extreme tiredness or back pain.

If you have any of the symptoms mentioned above, and they happen again and again, or they are new for you, it is important to see your doctor. If you have already been to your doctor and the symptoms have got worse or have not gone away, it is best to go back again and get checked out.

About Genetic Testing

To understand what a genetic test is, it first helps to understand what genes are.

Genes and inheritance Genes are made up of DNA. We each have about 20,000 genes, which act like a set of instructions, controlling our growth and how our bodies work. Genes are carried on 46 chromosomes which we inherit from our parents; 23 from our mother and 23 from our father. This means that we inherit two copies of each gene, one copy from each parent.

Genes and ovarian cancer Changes in genes are called mutations. Mutations are very common and we all carry a number of them. Sometimes we inherit or develop a mutation in a gene which stops it from working properly. In some cases this change causes a higher risk of developing certain cancers.

Changes in two genes, BRCA1 and BRCA2, which are 'cancer protection' genes if they work properly, cause an increased risk of developing breast and ovarian cancer. Changes in other

genes also increase the risk of developing ovarian cancer, bowel and womb cancer, but are not linked to breast cancer.

Genetic testing Genetic testing is done by taking some blood and looking at a person's genes.

Accuracy/Reliability Genetic testing takes place in a laboratory. Laboratory procedures, personnel, and quality control procedures must meet certain standards. Numerous steps are taken to ensure that the correct result is given. If a genetic mutation is found it is always double checked to ensure that the result is correct. Therefore, genetic tests are very accurate.

About PROMISE

Why is this research study important? Currently, it is difficult to detect ovarian cancer early. PROMISE has two aims: The first is early detection of ovarian cancer. Finding ovarian cancer earlier gives a better chance of survival and treatment. The second aim is prevention of ovarian cancer by stopping it from ever developing.

What is different about this research study? Existing cancer screening programmes like mammography offer screening to all women in the population who are over a certain age. PROMISE will use genetic information, alongside other information to see who is more or less likely to develop ovarian cancer. Screening is then offered to the women who have a higher chance of developing ovarian cancer.

Why is the study using genetic information? Most ovarian cancers are not caused by faulty genes *(Genes are the basic unit of heredity. They carry the information that determine the characteristics of a person)* and most women who get ovarian cancer do not have a family history of it. But a small number of ovarian cancers may be caused by a mutation (change) in a gene running in the family. Researchers have identified a number of genes which increase the risk of ovarian cancer.

Knowing which gene variants a woman carries gives a better estimate of personal ovarian cancer risk than not knowing. This means that options to manage the risk of ovarian cancer will be chosen to match the needs of each woman. Women who have a high risk may get more screening. They may also be offered surgery to reduce their risk. Women at low risk may be able to avoid unnecessary screening and other procedures. There are certain things that each woman can do to reduce her risk of ovarian cancer, no matter what her genetic risk.

Who can take part in the study? This study is available to women aged between 18 and 74.

How does PROMISE work? This new study will look at a woman's genes, personal health, and family history to estimate her lifetime risk of ovarian cancer. Her result will then be used to decide on the best way to manage her personal risk.

What information does the study give? Each woman will receive her personal risk of ovarian cancer based on the most up-to-date information. The estimate will be given in the form of 'one in…', for example 'one in 50'. Some people prefer to think of the risk as a percentage. A risk of one in 50 is the same as a 2% risk. These numbers tell us how likely it is that a woman will develop ovarian cancer in their lifetime.

Things to consider before taking part

It is your decision whether or not to have genetic testing *('A genetic test is used to look for changes in genes from a blood sample)* to learn about your personal lifetime risk of ovarian cancer. It may be helpful to weigh up potential benefits and harms before making a decision on whether or not to take part.

Benefits *Genetic testing will help to estimate personal risk of ovarian cancer*

This will allow health professionals to offer prevention and risk management options that are best suited to each individual woman.

The test result will give some information about breast cancer risk

There are some genes that increase the risk for both breast and ovarian cancer. Examples are the BRCA1 *(**Br**east **Ca**ncer Gene **1**.A cancer protection gene. Inherited changes in this gene increase cancer risk because it does not work properly)* and BRCA2 *('**Br**east **Ca**ncer Gene **2**. A cancer protection gene. Inherited changes in this gene increase cancer risk because it does not work properly)* genes. The new ovarian cancer screening programme will test these genes. This means you may receive information about your breast cancer risk. Because this study is about ovarian cancer, it will use the best possible way to predict ovarian cancer risks. It uses the best current way to predict breast cancer risk.

Potential harms *Receiving a high risk result may be upsetting*

Learning about your risk for ovarian cancer may be upsetting. Although getting a high risk result is rare, it is important to consider how knowing your genetic risk of ovarian cancer might make you feel. In response to a high risk result, some women may feel a range of emotions including shock, anger, worry and anxiety. Others may feel glad that they know. Some may feel a mixture of both. In the case of a high risk result, your GP will make an appointment with a genetic counsellor who you can talk to.

The genetic test will give some information about the risk of other women in your family

Genes are passed down from (biological) parents to their children. This is why family members of the same side share some genes. Your personal cancer risk may be similar to that of other women in your family because you share some genes. This means that other women in your family should be informed about your risk if it is high, so that they can get further testing. This can sometimes be difficult to manage, but genetic counsellors *(Genetic counsellors are health professionals that give help individuals and families with all aspects of genetic testing)* can help with the process.

The test can only tell about a woman's lifetime risk, not whether she has cancer right now

The genetic test will only tell about the risk of ovarian cancer over a woman's lifetime – not whether she has cancer right now. Therefore, it is important to know the symptoms of ovarian cancer, no matter whether or not you decide to take part in this research study.

Risk estimates aren't perfect

PROMISE can only give an estimate of ovarian cancer risk. Genes are only one factor that can influence on ovarian cancer risk. A high risk result does not mean that a woman will definitely get cancer, and a low risk does not mean that she definitely won't.

What are the options for managing high risk of ovarian cancer?

Screening Ovarian cancer screening aims to detect the cancer early so that it is easier to treat. This involves two different screening methods. One method is a blood test looking for a specific marker in the blood called CA125 *(CA125 is a protein found in the blood which may show that a woman has ovarian cancer)*. If the levels of CA125 rise significantly, this could mean that a woman may be developing ovarian cancer. The other method of screening is an ultrasound of your ovaries to see if a tumour is present. This ultrasound may be internal (transvaginal ultrasound) or external (transabdominal ultrasound).

Surgery Removing the ovaries using surgery aims to prevent the cancer from developing. This procedure is called a risk-reducing salpingo-oophorectomy (RRSO). Removing the ovaries will trigger the onset of the menopause *(Menopause is when the periods of a woman stop. This can happen naturally, or after surgery to remove the ovaries)*. There are options available to reduce the symptoms of the menopause. Every woman who has not had her menopause will be advised by her medical team on how to best manage this.

What are the possible results?

There are three possible outcomes of participating in this study.

1. You may learn that you are at LOW risk of developing ovarian cancer during your lifetime. Most women will have a low risk for ovarian cancer. Women in this group will have a very low chance of getting ovarian cancer during their lifetime. These women will not need regular screening *(Screening is testing individuals who have no symptoms to find cancer earlier)* for ovarian cancer. However, it is not impossible for these women to get ovarian cancer. Therefore, it is important to be aware of the symptoms of ovarian cancer, and to keep in mind that some lifestyle factors influence ovarian cancer risk.
2. You may learn that you are at INTERMEDIATE risk during your lifetime. About X in every X women are likely to be at intermediate risk of developing ovarian cancer in their lifetime. These women will be invited to see their GP to have further tests and discuss the best options to manage their risk.
3. You may learn that you are at HIGH risk of developing ovarian cancer during your lifetime. Few women will have a high risk. These women will be invited to see their GP to have further tests and discuss the best options to manage their risk. This may include an offer of surgery to have the ovaries removed.

Further information

Who is funding this study? This programme is currently jointly funded by Cancer Research UK and The Eve Appeal.

Will taking part in the study affect my life insurance? Sometimes people worry that if they have a genetic test they will have problems taking out life insurance. There is an agreed code of practice among insurance companies that results of genetic testing will not be used by the Association of British Insurers (ABI) to determine premiums or eligibility for life

insurance. Any genetic tests taken after the insurance policy has started do not have to be disclosed. This agreement was recently extended until 2017. Further information can be found on the website: www.abi.org.uk

I don't want to get a genetic test for risk of ovarian cancer. Can I still have ovarian cancer screening? No, unfortunately not. Without the genetic test, health professionals cannot tell whether ovarian cancer screening would carry more risks than benefits for you. Therefore, you will have to have a genetic test first.

Why do none of the other screening programmes use genetic testing? Although this is the first cancer screening study that includes genetic testing, it is likely that other cancer screening programmes will eventually also include genetic testing. For example, another research study is currently looking at genetic testing for breast cancer risk within the existing NHS Breast Screening Programme.

What can I do to reduce my risk of ovarian cancer? You may be able to reduce your risk of developing ovarian cancer by making changes to your lifestyle.

Lifestyle and prevention advice: Weight and smoking

Maintaining a healthy weight may help to reduce risk of ovarian cancer. Smoking may also increase the risk of ovarian cancer.

Using Oral Contraceptives ('The Pill')

Using the oral contraceptive pill for at least five years may reduce risk of ovarian cancer by up to 50%. We would not recommend you use the pill for this specific reason but it is a beneficial side effect.

Confidentiality Your participation in this study, and your results, will be completely confidential. Any information about yourself that you provide while taking part will be stored on a secure password protected database. This information will be treated as confidential and not released to anyone outside the research team working on this study.

Who are the doctors and researchers involved in this programme? This study is carried out by health professionals under the leadership of Professor Ian Jacobs and coordinated by University College London. We are also working with researchers from the University of Cambridge, University of Manchester, University of Southern California, Massachusetts General Hospital and Harvard University.
