# Supplementary material for: Impact of a decision aid about stratified ovarian cancer risk-management on women’s knowledge and intentions: a randomised online experimental survey study
Source: BMC Public Health. 2017 Nov 16;17:882. doi: 10.1186/s12889-017-4889-0 (PMC5689140; doi:10.1186/s12889-017-4889-0)
Supplement: Supplementary file 2 — Factor loadings for decisional conflict and anticipated decisional regret items; Table S2. Participants’ knowledge about genetics (‘genetic literacy’); Table S3. Participants’ knowledge about ovarian cancer and the PROMISE study in the sample overall. (DOCX 16 kb) [file 12889_2017_4889_MOESM2_ESM.docx]

**Supplemental Table 1.** Factor loadings based on a principle components analysis with varimax rotation from 8 items from the Decisional Conflict and Decisional Satisfaction Scales (N=1031)

|  | **Anticipated regret** | **Decisional conflict** |  |
| --- | --- | --- | --- |
| If I did not take part in PROMISE, I would feel regret | 0.91 | -0.06 |  |
| If I did not take part in PROMISE, I would later wish I had | 0.89 | -0.18 |  |
| I would be satisfied with my decision of whether or not to take part in PROMISE | -0.13 | 0.70 |  |
| I feel I would make an informed choice about taking part in PROMISE | -0.31 | 0.66 |  |
| I know the risks of taking part in PROMISE | -0.27 | 0.61 |  |
| I feel unsure of what I would choose - taking part in PROMISE or not taking part | -0.07 | -0.63 |  |
| The decision about whether or not to take part in PROMISE would be easy for me | -0.17 | 0.76 |  |
| I would expect to stick with my decision of whether or not to take part in PROMISE | -0.02 | 0.77 |  |
|  | **No. of items** | **Cronbach’s alpha** | ***M (SD)*** |
| Anticipated regret (possible range = 1-4) | 2 | 0.82 | 2.56 (0.66) |
| Decisional conflict (possible range = 1-4) | 6 | 0.45 | 2.09 (0.32) |

**Supplemental Table 2.** Participants’ knowledge about genetics (‘genetic literacy’)

| Genetic literacy item | Response options | N (%) | Mean score (SD) |
| --- | --- | --- | --- |
| Genes are inside cells (True) | **True**  Not true  Not sure | **636 (61.7)**  93 (9)  302 (29.3) |  |
| Genes are made up of DNA (True) | **True**  Not true  Not sure | **878 (85.2)**  44 (4.3)  109 (10.6) |  |
| Most genetic disorders are caused by a single gene (Not true) | True  **Not true**  Not sure | 446 (43.3)  **209 (20.3)**  376 (36.5) |  |
| If a person has a faulty gene that has been linked to a disorder, they will always get the disorder (Not true) | True  **Not true**  Not sure | 98 (9.5)  **688 (66.7)**  245 (23.8) |  |
| On average, a person has half their genes in common with their biological siblings (True) | **True**  Not true  Not sure | **587 (56.9)**  112 (10.9)  332 (32.2) |  |
| A mother and a daughter who look alike have more genes in common than a mother and a daughter who do not look alike (Not true) | True  **Not true**  Not sure | 138 (13.4)  **576 (55.9)**  317 (30.7) |  |
| **Total (range = 0-6)** |  |  | **3.47 (1.40)** |

**Supplemental Table 3.** Participants’ knowledge about ovarian cancer and the PROMISE study in the sample overall

|  |  | **Pre decision-aid** | **Post decision-aid** |
| --- | --- | --- | --- |
|  | **Response options** | **Total (n=1031)**  **N (%)** | **Total (n=1031)**  **N (%)** |
| *Knowledge about ovarian cancer:* |  |  |  |
| The average risk of a woman developing ovarian cancer in her lifetime is (1 in 50) | 1) 1 in 8  **2) 1 in 50**  3) 1 in 1000 | 285 (27.6)  **536 (52.0)**  210 (20.4) | 222 (21.5)  **693 (67.2)**  116 (11.3) |
| Ovarian cancer mostly affects older women (True) | **True**  Not true  Not sure | **191 (18.5)**  650 (63.0)  190 (18.4) | **457 (44.3)**  441 (42.8)  133 (12.9) |
| Ovarian cancer can easily be found early with an ultrasound scan (Not true) | True  **Not true**  Not sure | 471 (45.7)  **174 (16.9)**  386 (37.4) | 421 (40.8)  **271 (26.3)**  339 (32.9) |
| There are some factors which increase the risk of ovarian cancer. Please tick all that you think apply: |  |  |  |
| Being over 50 years old (Yes) | **Yes**  No | **435 (42.2)**  596 (57.8) | **662 (64.2)**  369 (35.8) |
| Being overweight (Yes) | **Yes**  No | **555 (53.8)**  476 (46.2) | **698 (67.7)**  333 (32.3) |
| Smoking (Yes) | **Yes**  No | **587 (56.9)**  444 (43.1) | **695 (67.4)**  336 (32.6) |
| Family history (Yes) | **Yes**  No | **856 (83.0)**  175 (17.0) | **858 (83.2)**  173 (16.8) |
| Taking the pill (No) | Yes  **No** | 394 (38.2)  **637 (61.8)** | 312 (30.3)  **719 (69.7)** |
| Having children (No) | Yes  **No** | 125 (12.1)  **906 (87.9)** | 103 (10.0)  **928 (90.0)** |
| Breastfeeding (No) | Yes  **No** | 25 (2.4)  **1006 (97.6)** | 28 (2.7)  **1003 (97.3)** |
| Total mean (range = 0-10) (SD) |  | 5.71 (1.55) | 6.77 (1.82) |
| *Knowledge about PROMISE:* |  |  |  |
| Genetic testing for ovarian cancer involves giving a blood sample (True) | **True**  Not true  Not sure | n/a | **561 (54.4)**  136 (13.2)  334 (32.4) |
| PROMISE uses genetic information to estimate the risk of developing ovarian cancer (True) | **True**  Not true  Not sure | n/a | **717 (69.5)**  50 (4.8)  264 (25.6) |
| Learning about personal risk of ovarian cancer may affect other family members (True) | **True**  Not true  Not sure | n/a | **775 (75.2)**  99 (9.6)  157 (15.2) |
| Most women who take part in PROMISE will learn that they are at high risk of ovarian cancer (Not true) | True  **Not true**  Not sure | n/a | 144 (14.0)  **656 (63.6)**  231 (22.4) |
| There is nothing that women who are at high risk of ovarian cancer can do to reduce their risk of ovarian cancer (Not true) | True  **Not true**  Not sure | n/a | 109 (10.6)  **650 (63.0)**  272 (26.4) |
| Women who take part in PROMISE will get some information about their risk of breast cancer (True) | **True**  Not true  Not sure | n/a | **733 (71.1)**  42 (4.1)  256 (24.8) |
| Total mean (range = 0-6) (SD) |  | n/a | 3.97 (1.72) |
